# Women's quality of life is decreased by acute cystitis and antibiotic adverse effects associated with treatment

**DOI:** 10.1186/1477-7525-3-45

**Published:** 2005-07-27

**Authors:** Erika J Ernst, Michael E Ernst, James D Hoehns, George R Bergus

**Affiliations:** 1 College of Pharmacy, University of Iowa, Iowa City, IA, USA; 2 Department of Family Medicine, University of Iowa Health Care, Iowa City, IA, USA; 3 Carver College of Medicine, University of Iowa, Iowa City, IA, USA; 4 Northeast Iowa Medical Education Foundation, Waterloo, IA, USA

## Abstract

**Background:**

Although acute cystitis is a common infection in women, the impact of this infection and its treatment on women's quality of life (QOL) has not been previously described.

Objectives: To evaluate QOL in women treated for acute cystitis, and describe the relationship between QOL, clinical outcome and adverse events of each of the interventions used in the study.

**Methods:**

Design. Randomized, open-label, multicenter, treatment study.

Setting. Two family medicine outpatient clinics in Iowa.

Patients. One-hundred-fifty-seven women with clinical signs and symptoms of acute uncomplicated cystitis.

Intervention. Fifty-two patients received trimethoprim/sulfamethoxazole 1 double-strength tablet twice daily for 3 days, 54 patients received ciprofloxacin 250 mg twice daily for 3 days and 51 patients received nitrofurantoin 100 mg twice daily for 7 days.

Measurements. QOL was assessed at the time of enrollment and at 3, 7, 14 and 28 days after the initial visit. QOL was measured using a modified Quality of Well-Being scale, a validated, multi-attribute health scale. Clinical outcome was assessed by telephone interview on days 3, 7, 14 and 28 using a standardized questionnaire to assess resolution of symptoms, compliance with the prescribed regimen, and occurrence of adverse events.

**Results:**

Patients experiencing a clinical cure had significantly better QOL at days 3 (p = 0.03), 7 (p < 0.001), and 14 (p = 0.02) compared to patients who failed treatment. While there was no difference in QOL by treatment assignment, patients experiencing an adverse event had lower QOL throughout the study period. Patients treated with ciprofloxacin appeared to experience adverse events at a higher rate (62%) compared to those treated with TMP/SMX (45%) and nitrofurantoin (49%), however the difference was not statistically significant (p = 0.2).

**Conclusion:**

Patients experiencing cystitis have an increase in their QOL with treatment. Those experiencing clinical cure have greater improvement in QOL compared to patients fail therapy. While QOL is improved by treatment, those reporting adverse events have lower overall QOL compared to those who do not experience adverse events. This study is important in that it suggests that both cystitis and antibiotic treatment can affect QOL in a measurable way.

## Background

Acute uncomplicated cystitis is a common community acquired infection in women, causing an estimated 7 million episodes yearly in the United States [1]. On average, each episode is associated with 1.2 days of missed work or classes [1]. The cost of treating ambulatory patients with symptoms of dysuria is estimated to exceed 1 billion dollars per year in the United States [1].

As with the majority of infectious diseases, empiric therapy is the cornerstone of treatment for acute uncomplicated cystitis. When obtained, urine cultures often take several days for results, making it necessary to begin therapy empirically. Selection of the antibiotic must take into consideration many factors, including results of clinical trials demonstrating efficacy of a chosen regimen, known susceptibility patterns of commonly infecting organisms, the clinical condition of the patient, as well as drug allergies, rate of adverse reactions and cost of treatment. In many settings, particularly health maintenance organizations, treatment algorithms exist for the empiric treatment of acute cystitis [2,3]. In most cases, these guidelines suggest treatment based upon symptoms of disease in otherwise healthy adult women and recommend against obtaining culture and sensitivity tests [2]. At least one study has shown this treatment strategy to be the most cost-effective means of therapy [4].

In order to fully compare potential antimicrobial regimens, factors such as clinical cure rates, complications, including adverse events of treatment, and quality of life (QOL) should be taken into account. While there are several tools available in the literature to evaluate QOL, the majority are applied to patients with chronic diseases such as rheumatoid arthritis or with cancer [5]. One pilot study has been published evaluating QOL in acute cystitis and found that it is significantly affected by the illness [6]. However, the impact of treatment on QOL has not been evaluated in the previously published study.

Additionally, the evaluation of adverse effects related to different treatments and the impact on QOL have not been described previously for cystitis. Most of the published literature comparing the impact of treatment on a patients' QOL is limited to chronic diseases or treatments with severe adverse effects such as cancer chemotherapy [7-10]. We sought to determine the impact of a common infection, cystitis, on QOL and evaluate the impact of three different treatment regimens on QOL in these patients. The addition of QOL information could be useful to primary care physicians when deciding on an antibiotic for empiric treatment of cystitis.

## Methods

Patients were recruited from two outpatient family medicine clinics in the state of Iowa, both affiliated with the University of Iowa College of Medicine and College of Pharmacy. Women aged 18 to 65 were eligible for inclusion in the study if they had clinical symptoms of cystitis, including dysuria or frequency, and were considered by their primary physician to have acute uncomplicated cystitis and were not allergic to any study medication. Patients were excluded if they had diabetes, known anatomical abnormalities, urinary symptoms for greater than 7 days, vaginal discharge, or were pregnant. These exclusion criteria were intended to select for acute cystitis patients and exclude patients who may have pyelonephritis or sexually transmitted diseases. All study procedures were approved by the Institutional Review Board at the University of Iowa.

After obtaining written informed consent, patients provided a urine specimen for culture and sensitivity. Patients were then randomized, using a random number generator, to one of three treatments; a) Trimethoprim/Sulfamethoxazole (TMP/SMX) 800 mg/160 mg (Qualitest Pharmaceuticals, Inc, Huntsville, AL) 1 tablet twice daily for 3 days, b) ciprofloxacin 250 mg (Cipro, Bayer Corporation, West Haven CT) 1 tablet twice daily for 3 days or c) nitrofurantoin 100 mg (Macrobid, Proctor & Gamble Pharmaceuticals, Cincinnati, OH) 1 capsule twice daily for 7 days. Nitrofurantoin was given for seven days because clinical studies have shown it to be less effective in a three day regimen compared to TMP/SMX [11]. Medication was provided to the patient by a pharmacist on the research team.

Outcome was assessed by telephone interview using a standardized questionnaire to assess resolution of symptoms, compliance with the prescribed regimen, and occurrence of adverse events. Patients were questioned using a prompted list of symptoms and also asked if they experienced any adverse or side effects from the prescribed regimen. QOL was measured using the Quality of Well-Being, a validated, multi-attribute health scale [12]. This scale was selected because it has been successfully applied to acute illnesses, whereas other quality of life scales, such as the SF-36 Health Survey, are better suited to assess chronic illnesses [4]. Higher scores on the Quality of Well-Being reflect higher QOL. The questionnaire was modified slightly to be administered over the telephone and was re-administered by a trained interviewer at each follow up contact. Four follow-up telephone contacts were necessary to evaluate early and late recurrence of cystitis, and were completed at 3, 7, 14 and 28 days after the initial visit.

Clinical cure was defined as the absence of symptoms and no additional antibiotic treatment for urinary symptoms within the 28 day follow up period. Therefore, if a woman reported continued symptoms at day 7 but did not receive additional antibiotic treatment and had resolution of symptoms on follow up at days 14 and 28, she was considered a clinical cure. These criteria were selected with the purpose of mimicking usual care. Urine culture results were classified as positive (greater that 50,000 colony forming units per milliliter), negative (less than 50,000 colony forming units per milliliter) or contaminated (containing multiple organisms at less than 50,000 colony forming units per milliliter).

The two tailed t-test for independent samples was used to compare QOL by outcome and adverse events. One-way analysis of variance with the Bonferroni correction was used to compare QOL by culture results (positive, negative, contaminated) or drug treatment (TMP/SMZ, Ciprofloxacin, Nitrofurantoin). Differences in cure rates between treatments were compared using the chi square test. Fisher's exact test was used to compare outcome and organism susceptibility. We estimate power of 75% to detect significant differences in QOL, adverse events and outcome at the alpha level of 0.05.

## Results

### Patient enrolment and demographics

**Figure 1:**
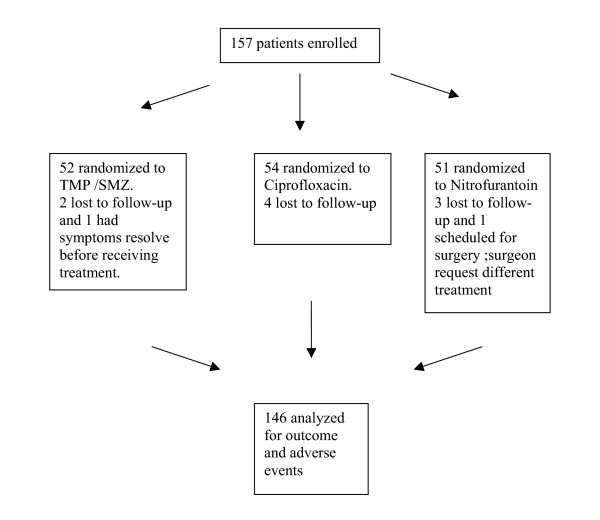
Diagram of patient enrolment and analysis.

One-hundred-fifty-seven patients met the inclusion criteria and were enrolled in the study. Eleven patients were excluded from the analysis of outcome and adverse events. Of these 11, nine were lost to follow up, one had her symptoms resolve before she took the medication and one was scheduled for surgery and her surgeon wanted her to receive a different medication. One patient experienced severe nausea after one day and was prescribed another antibiotic. This patient was counted as experiencing an adverse event and a clinical failure. See figure 1 for the diagram of patient enrollment.

The mean age of women enrolled in the study was 34 years (range 18–69). There were no significant differences in baseline demographic between the randomized groups (Table 1). Of the 157 women enrolled, 57% had positive urine cultures, 26% had negative cultures, and 17% had contaminated specimens. E. coli accounted for 82% of the positive urine cultures (Table 2). Of all organisms isolated, resistance to TMP/SMZ, ciprofloxacin and nitrofurantoin were 7%, 1% and 8%, respectively. For E. coli the resistance rates were 7%, 0% and 4% for TMP/SMX, ciprofloxacin and nitrofurantoin, respectively.

**Table 1:** Patient Demographics

	**All Patients**	**TMP/SMZ**	**Ciprofloxacin**	**Nitrofurantoin**
Age in years (mean ± SD)	34 ± 12	33 ± 12	32 ± 12	36 ± 12
Weight in kg (mean ± SD)	73 ± 21	73 ± 22	72 ± 22	74 ± 22
Overweight, >20% of ideal body weight (%)	26	29	22	31
Number of concomitant medications (mean ± SD, range)	1 ± 2, 0–12	1 ± 2	1 ± 2	1 ± 2
Received antibiotics in previous 6 months (%)	34	35	30	38
Receiving oral contraceptives or hormone replacement therapy (%)	39	33	48	35
Sexually active (%)	85	89	85	80
Urinary tract infection in previous 6 months (%)	37	40	43	28

**Table 2:** Organism species and susceptibility results of culture specimens from women with acute uncomplicated cystitis

	**N**	**%**	**% susceptible TMP/SMX**	**Ciprofloxacin**	**Nitrofurantoin**
***E. coli***	73	82	93	100	95
***P. mirabillis***	5	5.6	100	100	na
***K. pneumonia***	4	4.4	100	100	50
***S. aureus***	3	3.3	100	0	100
***C. koseri***	2	2.2	100	100	100
***B-hemolytic Strep***	1	1.1	na	na	na
***S. saphophyticus***	1	1.1	0	100	100
**Total**	89	100			

na = not available

### Clinical outcomes

Clinical cure rates did not differ by treatment with 88, 82 and 87% of patients treated with TMP/SMX, ciprofloxacin and nitrofurantoin, respectively experiencing a clinical cure (p = 0.7). Patients randomized to TMP/SMX who were infected with a resistant organism had lower cure rates, compared to those infected with a susceptible organism (50% vs. 83%), although this difference was not statistically significant due to the low number of infections with resistant organisms (Table 3). Patients treated with ciprofloxacin appeared to experience adverse events at a higher rate (62%) compared to those treated with TMP/SMX (45%) and nitrofurantoin (49%); however, the difference was also not statistically significant (p = 0.2).

**Table 3:** Percent (no.) of patients successfully treated by organism susceptibility

	**Susceptible**	**Resistant**	**p value**
TMP/SMZ	83% (43/52)	50% (1/2)	0.9
Ciprofloxacin	76% (41/54)	---	--
Nitrofurantoin	80% (41/51)	100% (1/1)	0.6

### Quality of life outcomes

**Figure 2:**
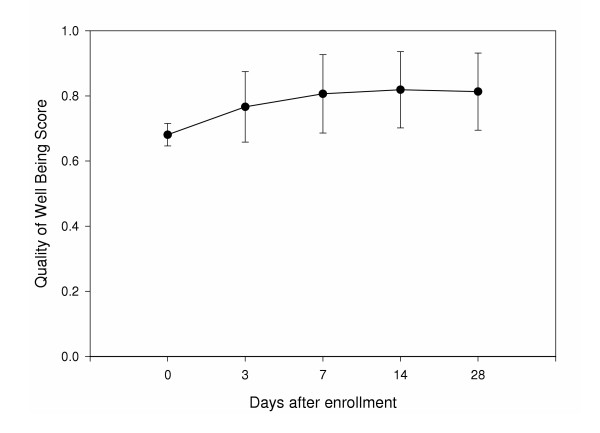
Quality of Life for all patient at each follow up contact.

**Figure 3:**
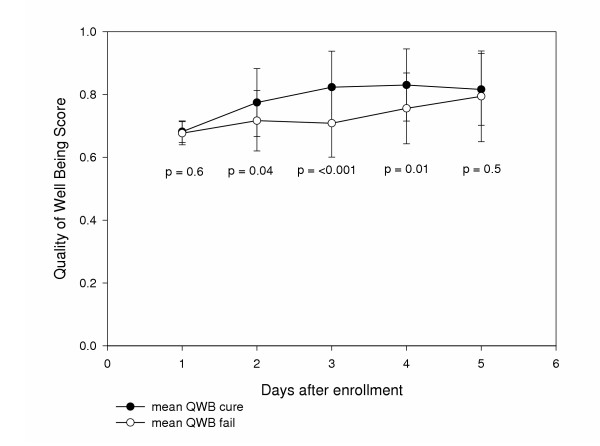
Quality of Life in patients by clinical outcome at each follow up contact.

**Figure 4:**
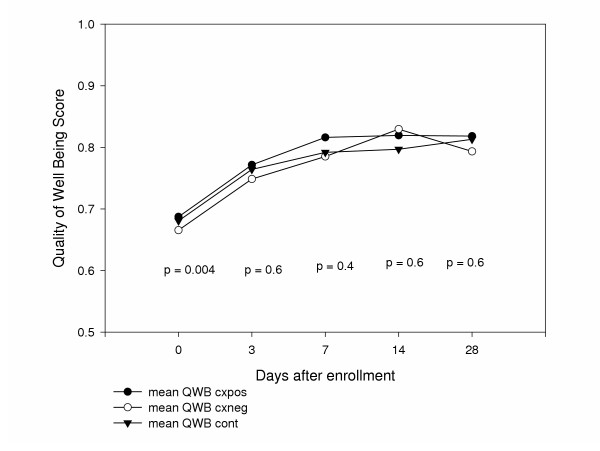
Quality of Life in patients by urine culture results at each follow up contact.

**Figure 5:**
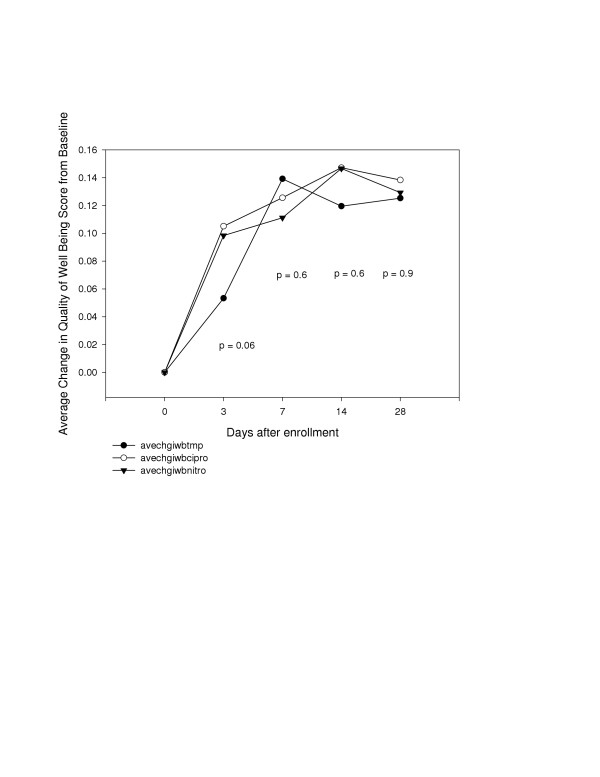
Change in quality of life by drug treatment over the study period.

Overall, the QOL of patients treated for acute uncomplicated cystitis improved over the study period. Quality of Well Being scores improved for all patients, from a mean (± SD) of 0.68 (± 0.03) at baseline to 0.81 (± 0.11) at day 28 (Figure 2). Patients experiencing a clinical cure had significantly better QOL at days 3 (0.77 vs. 0.72, p = 0.04), 7 (0.82 vs. 0.71, p < 0.001), and 14 (0.83 vs. 0.76, p = 0.01) compared to patients who failed treatment (Figure 3). QOL at baseline was not different between patients successfully treated compared to those who failed therapy (0.68 vs. 0.68, p = 0.6) or day 28 (0.82 vs. 0.79, p = 0.5). There was a statistically significant difference in QOL by culture result (p = 0.004). In the post hoc analysis, patients with positive urine cultures had significantly higher quality of well being scores compared to patients with negative urine cultures (0.69 versus 0.66, p = 0.003) but not compared to patients with contaminated urine cultures (0.68, p = 0.23) at baseline. All three groups had improvement in their QOL over the study period (Figure 4). There was no significant difference in improvement of QOL by treatment assignment (Figure 5).

### Adverse effects

**Figure 6:**
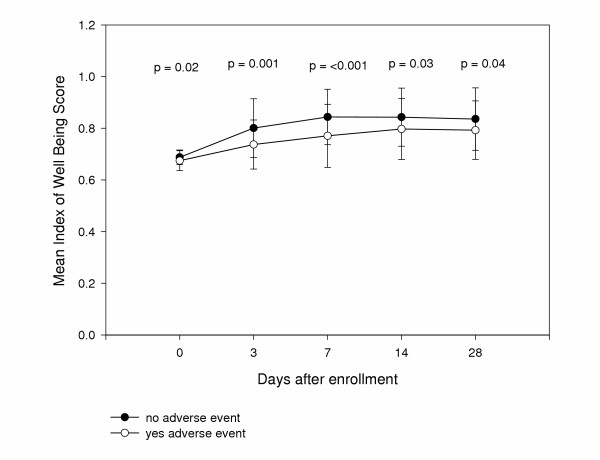
Patient quality of life by adverse event experience at each follow up contact.

Seventy-six of the 146 patients included in the analysis reported 94 adverse events. Gastrointestinal effects and vaginitis were the most commonly occurring adverse events, accounting for 26 (28%) and 25 (27%) of the effects, respectively. Headache (12% of patients) and other central nervous system effects such as dizziness (5%) were the next most common side effects. The remaining 12% of adverse events were a variety of miscellaneous complaints. Patients experiencing an adverse event had significantly lower QOL compared to those not experiencing an adverse event throughout the study period, including differences at baseline (Figure 6).

## Discussion

We have described the impact of a seemingly minor infection, acute uncomplicated cystitis, on the QOL of women. Our study is the first to examine the effect of successful treatment compared to treatment failure on the QOL. Further, we have also shown that experiencing an adverse effect from treatment also directly impacts QOL. While the three antibiotics used in this study differed in terms of cure rates and adverse effects we found little difference in the impact on QOL between treatments.

Current recommendations suggesting empiric therapy with TMP/SMX without obtaining cultures may need to be modified in the era of increasing resistance [1,3,4,13]. However, altering empiric therapy recommendations to either nitrofurantoin or fluoroquinolone antibiotics, without information on local resistance patterns, may result in unnecessary increases in health care expenditures due to increased drug costs. In primary care offices, where limiting the inappropriate prescribing of antimicrobial therapy is receiving focused attention by the Centers for Disease Control and Prevention, information regarding the emergence of resistance and clinical outcome is essential to providing evidence-based recommendations for empiric antimicrobial therapy for acute uncomplicated cystitis.

In our study, we found the resistance rate of E. coli to TMP/SMX remained low, approximately 7% for women with cystitis. We also found that clinical cure did not differ between the treatment regimens tested. These findings are important because it means TMP/SMX continues to be an effective first line antibiotic in our population.

Our findings support those of a previously published study, indicating that uncomplicated cystitis has a significant, measurable impact on patients' QOL [6]. Patients' QOL was improved throughout the study period (figure 2). QOL was significantly higher in patients experiencing a clinical cure compared to those who failed treatment at days 3, 7 and 14 (figure 3). QOL did not differ between these groups at baseline and 28 days. This indicates all patients were experiencing significant decreases in QOL at baseline. It also indicates we are able to measure the impact of cystitis on QOL. The fact that QOL was not different between patients experiencing a cure or failure of initial therapy, reflects the success of alternate treatment after experiencing the initial failure.

QOL at baseline was not different between patients with positive or contaminated urine cultures. However, QOL did differ significantly at baseline between patients with positive and negative urine cultures. One possible explanation for this difference is that patients with culture negative cystitis are more sensitive to the impact of cystitis symptoms on their QOL compared to those with positive urine cultures. That is to say that while their urine culture was negative, the urinary symptoms they were experiencing had a greater impact on QOL compared with patients that have a higher overall QOL. Alternatively, patients with culture negative cystitis may differ in some other way that impacts their QOL compared to patients with culture positive cystits.

We did not find any significant difference between the drug treatments on patients' QOL throughout the study period (figure 5). While there was a trend toward lower QOL in the group of patients treated with TMP/SMZ at day 3, this difference did not reach statistical significance (p = 0.06). Therefore based upon improvements in QOL, all three treatments may be considered appropriate empirical therapy for cystitis.

Previously, the effect of medication adverse events on QOL has only been demonstrated with more severe adverse events such as those experienced with cancer chemotherapy [14-16]. We have shown that even common side effects of antibiotics such as gastrointestinal effects have a negative impact on patients QOL. While some difference was observed in the rate of adverse events reported between treatment groups, this difference did not reach statistical significance. We also found that patients reporting adverse events had lower QOL throughout the study period, including baseline and at 28 days. This may suggest that patients with lower overall QOL may be more sensitive to the addition of drug therapy to their daily routine. The potential for patients having lower QOL reporting more adverse events has not been previously described. This information along with our findings that patients with negative urine cultures also have lower QOL suggests certain patients experience greater impacts on their QOL from seemingly innocuous occurrences such as urinary symptoms of cystitis or taking antibiotics compared to other patients. It is possible that health care providers may need to approach diagnosis and treatment in these patients differently from other patients. More care may be necessary in evaluating symptoms and laboratory information to decide whether treatment is necessary and more care may be needed in drug therapy selection, for example.

## Conclusion

In summary, we have found that patients experiencing cystitis have significant decreases in QOL and that QOL is improved by effective treatment. Furthermore we have found that patients reporting adverse events have lower overall QOL and may be impacted to a greater extent by simple infections and adverse events associated with treatment.

## Authors' contributions

EJE conceived the study, participated in its design and coordination, performed the statistical analysis and drafted the manuscript. MEE participated in the study design, coordination and patient recruitment and helped revise the manuscript. JDH participated in patient recruitment and helped revise the manuscript. GRB participated in study design, data analysis and manuscript revision. All authors read and approved the final manuscript.
